# Leucine-Rich Alpha-2 Glycoprotein 1 Accumulates in Complicated Atherosclerosis and Promotes Calcification

**DOI:** 10.3390/ijms242216537

**Published:** 2023-11-20

**Authors:** Lucile Grzesiak, Ana Amaya-Garrido, Guylène Feuillet, Nicole Malet, Audrey Swiader, Marie-Kerguelen Sarthou, Amandine Wahart, Damien Ramel, Stéphanie Gayral, Joost Peter Schanstra, Julie Klein, Muriel Laffargue

**Affiliations:** 1 Institut National de la Santé et de la Recherche Médicale (INSERM), U1297, Institute of Cardiovascular and Metabolic Disease, 31432 Toulouse, France; 2 Department of Biology, Université Toulouse III Paul-Sabatier, 31062 Toulouse, France

**Keywords:** atherosclerosis, vascular smooth muscle cell, calcification, LRG1

## Abstract

Atherosclerosis is the primary cause of cardiovascular disease. The development of plaque complications, such as calcification and neo-angiogenesis, strongly impacts plaque stability and is a good predictor of mortality in patients with atherosclerosis. Despite well-known risk factors of plaque complications, such as diabetes mellitus and chronic kidney disease, the mechanisms involved are not fully understood. We and others have identified that the concentration of circulating leucine-rich α-2 glycoprotein 1 (LRG1) was increased in diabetic and chronic kidney disease patients. Using apolipoprotein E knockout mice (ApoE−/−) (fed with Western diet) that developed advanced atherosclerosis and using human carotid endarterectomy, we showed that LRG1 accumulated into an atherosclerotic plaque, preferentially in calcified areas. We then investigated the possible origin of LRG1 and its functions on vascular cells and found that LRG1 expression was specifically enhanced in endothelial cells via inflammatory mediators and not in vascular smooth muscle cells (VSMC). Moreover, we identified that LRG1 was able to induce calcification and SMAD1/5-signaling pathways in VSMC. In conclusion, our results identified for the first time that LRG1 is a direct contributor to vascular calcification and suggest a role of this molecule in the development of plaque complications in patients with atherosclerosis.

## 1. Introduction

Atherosclerosis is a chronic inflammatory disease leading to the development of cardiovascular complications over time. The most advanced complications include reduced blood flow caused by plaque protrusion in the vessel or complete occlusion due to a thrombus, resulting in stroke or myocardial infarction [1]. Therefore, preventing and predicting plaque rupture is crucial for the clinical management of patients with cardiovascular disease. Advanced and unstable plaques are defined by a marked inflammation, a large lipid core, decreased vascular smooth muscle cells (VSMC), a thin fibrous cap, and intra-plaque hemorrhage [2,3]. Another marker of advanced plaques is calcification; however, its role in plaque rupture remains under investigation [4,5]. While microcalcifications appear to be associated with plaque instability [4,6,7,8], macrocalcifications are thought to stabilize the plaque [5,9,10]. It has been demonstrated that calcification development is aggravated by known risk factors, such as diabetes mellitus and chronic kidney disease, and that calcification is associated with increased arterial stiffness [11,12] and the development of cardiovascular complications in patients with chronic kidney disease and diabetes mellitus [13]. Therefore, it is essential to better understand the molecular mechanisms underlying calcification to propose novel therapeutic strategies.

Vascular calcification is characterized as the ectopic deposition of calcium and phosphate crystals in the vessel wall. This process involves a complex interplay of factors promoting or inhibiting calcification along with the dysregulation of inflammation, oxidative stress, and apoptosis in vascular cells [14,15]. Typically, calcification is triggered by the trans-differentiation of VSMC into osteoblast-like cells, and it is primarily regulated by the TGFβ/BMP superfamily signaling pathways. This phenotype is marked by the upregulation of osteochondrogenic transcription factors, such as runt-related transcription factor 2 (Runx2) or osteopontin (Spp1), which promote calcification [16]. Pro-inflammatory cytokines, like interleukin 1 β (IL1β) or tumor necrosis factor α (TNFα) [16], which are abundant in atherosclerotic plaques [17], induce the VSMC trans-differentiation. 

Interestingly, we and others have identified elevated levels of leucine-rich α-2 glycoprotein 1 (LRG1) in the plasma of diabetic and chronic kidney disease patients [18,19]. LRG1 is a glycoprotein that belongs to the leucine-rich repeat protein family and is capable of binding different receptors, such as the TGFβ receptors ALK1 and ALK5, and the TGFβ co-receptor endoglin, to modulate the TGFβ signaling pathway [20,21]. Ample evidence demonstrates that LRG1 expression may be affected by inflammatory conditions and is associated with the development of cardiovascular complications [19,22,23]. Despite this evidence, the possible involvement of LRG1 in vascular calcification has never been explored. 

Therefore, the aim of this study was to investigate LRG1 in advanced atherosclerosis and calcification. Our results indicate that LRG1 accumulates in the neointima of advanced calcified plaques in both mice and humans. Additionally, we demonstrated that LRG1 was able to promote trans-differentiation of VSMC. This suggests that LRG1 may not only serve as a prognostic marker for cardiovascular complications but may also play a role in arterial calcification. Our findings provide novel insights into the molecular mechanisms of the intimal calcification of VSMC induced by LRG1.

## 2. Results

### 2.1. LRG1 Is Localized in Calcified Regions of Atherosclerotic Plaques in Mouse and Human

**Figure 1:**
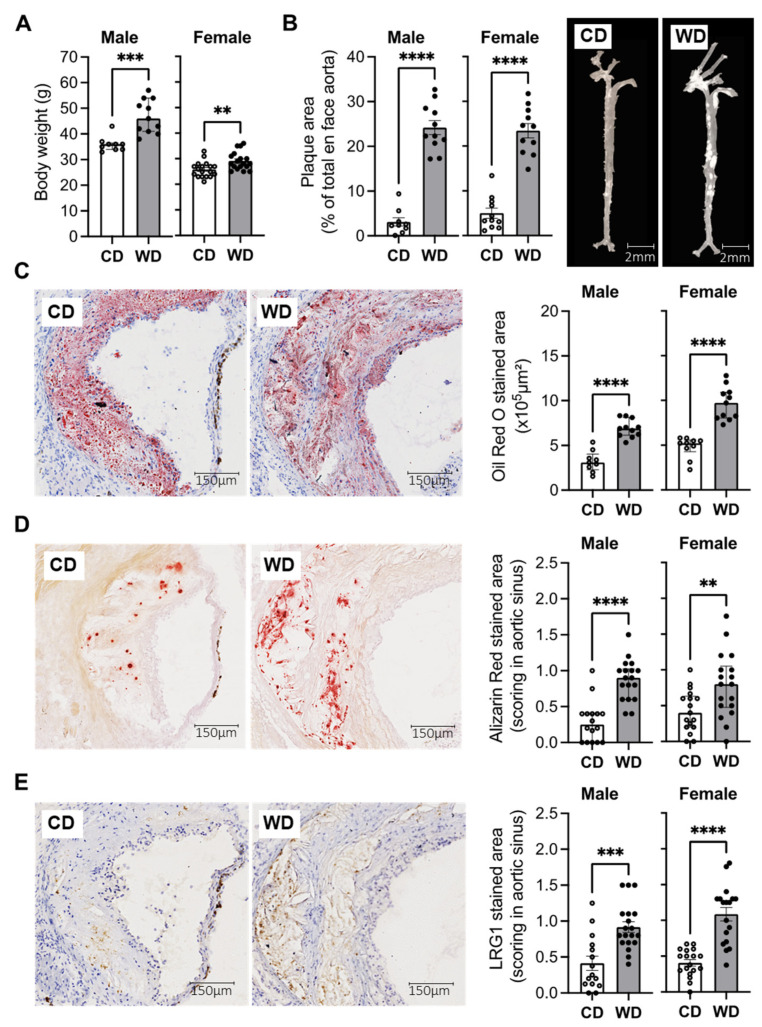
LRG1 in calcified atherosclerotic plaques in a mouse model of advanced atherosclerosis. (**A**) Body weights in male (*n* = 9–11/group) and female (*n* = 18/group) ApoE−/− mice after 27 weeks of Western diet (WD) or normal chow diet (CD). (**B**) Quantification of the plaque area and representative images of en face aortas for CD and WD-fed male and female mice (*n* = 9–11/group). (**C**–**E**) Representative histological images and quantitative analysis in serial sections of aortic sinus from CD and WD-fed male and female mice showing plaque lipid content with Oil Red O coloration (*n* = 9–11/group) ((**C**) red staining), plaque calcification with the Alizarin Red staining (*n* = 16–18/group) ((**D**) dark red staining) and LRG1 expression (*n* = 16–18/group) ((**E**) brown staining). Scoring for Alizarin Red and LRG1 was performed with 3 scores: 0 (no staining), 1 (weak staining), and 2 (strong localized staining). ** *p* < 0.01, *** *p* < 0.001, **** *p* < 0.0001 via Mann–Whitney U test for (**A**,**C**,**D**) and Student’s *t*-test for (**B**,**E**). Normally distributed data are presented as mean ± SEM, and non-normally distributed data are presented as median with lower and upper quartiles.

In order to study the role of LRG1 in the development of plaque complications, we used a mouse model of advanced atheroma using male and female apolipoprotein E knockout mice (ApoE−/−) fed a Western diet (WD) for 27 weeks. Compared with mice fed with chow diet (CD), the WD-fed mice presented increased body weight (Figure 1A) and extensive atherosclerotic lesions across the entire aorta as demonstrated in en face analysis (Figure 1B).

Serial cryosections of CD and WD-fed mouse aortic sinus were next analyzed for their lipid content and the presence of plaque calcification. In both male and female mice, the WD-fed group developed more extensive atherosclerotic lesions compared to the CD-fed group, as illustrated in the Oil Red O staining (Figure 1C). The plaques in the WD-fed group presented increased calcification levels compared with the CD-fed group, as shown with a higher Alizarin score (Figure 1D). We then observed a marked increase in LRG1 expression in complicated plaques in the WD-fed group compared to the intermediate lesions in the CD-fed group (Figure 1E). Furthermore, LRG1 expression was localized in the same regions of the plaque as the Alizarin staining, occurring around microcalcifications, especially accumulating in larger punctate calcifications (Figure 1D,E). 

**Figure 2:**
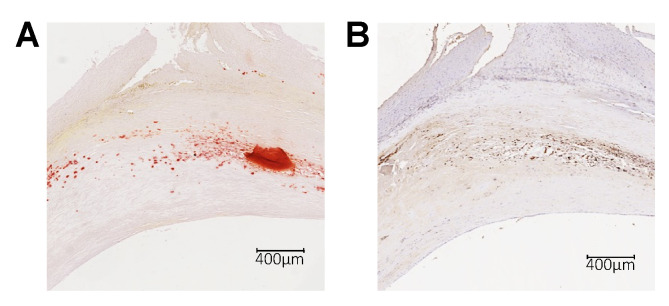
LRG1 in calcified atherosclerotic plaques in humans. Histological images of human carotid plaque serial sections showing (**A**) plaque calcification with the Alizarin Red staining (dark red staining) and (**B**) LRG1 expression (brown staining).

To confirm these observations in humans, we analyzed LRG1 presence in human atherosclerotic plaque obtained from carotid endarterectomy. As observed in mice, LRG1 staining localized in the Alizarin-positive area demonstrating that LRG1 is preferentially localized in calcified areas of atherosclerotic plaque (Figure 2). Therefore, these results suggest that LRG1 is expressed in complicated plaques, especially in calcified regions.

### 2.2. LRG1 Expression Is Induced in Endothelial Cells by Pro-Inflammatory Cytokines

To better understand how LRG1 accumulates in the atherosclerotic plaque, we investigated the vascular origin of LRG1 expression. 

**Figure 3:**
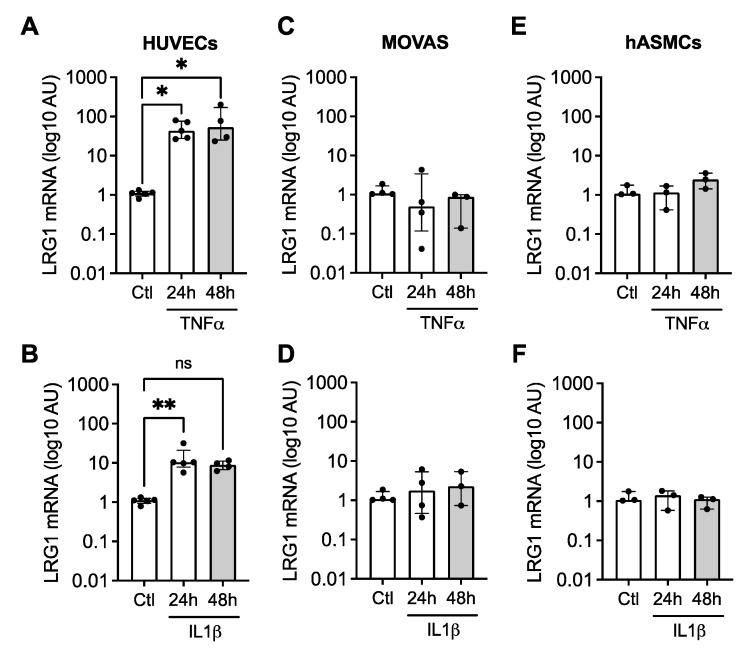
LRG1 expression in endothelial cells and VSMC in response to inflammatory stimuli. Quantification of LRG1-relative mRNA expression in (**A**,**B**) human umbilical vein endothelial cells HUVECs, (**C**,**D**) murine vascular smooth muscle cells MOVAS, and (**E**,**F**) human vascular smooth muscle cells hASMCs stimulated for 24 h and 48 h with 30 ng/mL of TNFα or 4 ng/mL of IL1β. The expression levels of LRG1 mRNA were corrected with Rpl13 expression. N = 3–5 independent experiments/group; AU: Arbitrary Unit; * *p* < 0.05, ** *p* < 0.01 by Kruskal–Wallis with Dunn’s multiple comparison test. Data are presented as median with lower and upper quartiles.

We analyzed the mRNA expression of LRG1 in endothelial cells (human umbilical vein endothelial cells, HUVEC) and in murine (MOVAS) and human (human aortic smooth muscle cells, hASMC) VSMC, which were stimulated for 24 h and 48 h with TNFα or IL1β, two major pro-inflammatory cytokines involved in atherosclerosis. We observed that HUVEC cells expressed higher LRG1 mRNA levels after treatment with TNFα or IL1β (Figure 3A,B). Neither mouse nor human VSMC showed any increase in LRG1 mRNA in response to these cytokines (Figure 3C–F), suggesting that LRG1 originates from activated endothelium during atherosclerosis.

### 2.3. LRG1 Promotes VSMC Trans-Differentiation and Calcification

**Figure 4:**
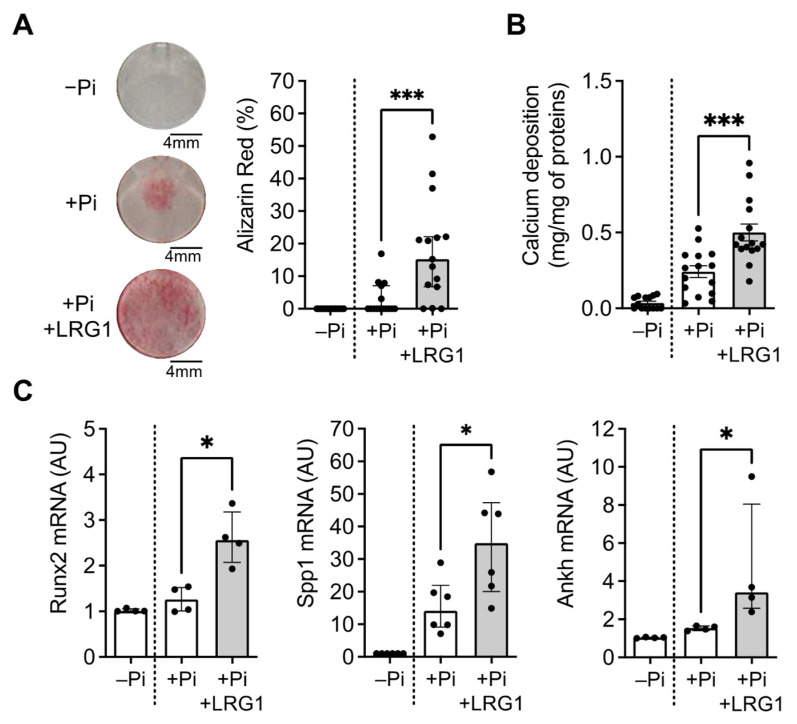
Effect of LRG1 in VSMC trans-differentiation and calcification. (**A**,**B**) Representative images and quantification of Alizarin Red staining (**A**) and quantification of calcium deposition (**B**) in MOVAS treated with control (−Pi) or calcification medium (+Pi) with or without 20 μg/mL LRG1 (+LRG1) for 6 days (*n* = 15 replicates/group from 5 independent experiments). (**C**) Quantification of osteoblast-like cells phenotype markers relative mRNA expression Runx2, Spp1, and Ankh in MOVAS treated with control (−Pi) or calcification medium (+Pi) with or without 20 μg/mL LRG1 (+LRG1) for 6 days (*n* = 4 independent experiments/group). * *p* < 0.05, *** *p* < 0.001 by Mann–Whitney U test for (**A**,**C**) and Student’s *t*-test for (**B**). Normally distributed data are presented as mean ± SEM and non-normally distributed data are presented as median with lower and upper quartiles.

Knowing that LRG1 was localized in the calcified regions of the plaque, we next evaluated its possible involvement with vascular calcification. MOVAS cells were cultivated in a calcification medium containing a phosphate buffer (+Pi) or in a control medium (−Pi) and were stimulated with LRG1 for 6 days. We observed that LRG1 significantly induced VSMC calcification, as shown in the increased Alizarin staining (Figure 4A) and calcium deposition (Figure 4B), compared to the calcification medium alone. We next measured the mRNA expression of markers VSMC trans-differentiation into osteoblast-like cells in the same conditions. Expression levels of Runx2, Spp1, and Ankh (progressive ankylosis protein) were increased in MOVAS after stimulation with LRG1 compared with the calcification medium alone (Figure 4C). These results indicate that LRG1 could be a direct inducer of VSMC calcification. 

### 2.4. LRG1 Potentiates TGFβ-Induced SMAD1/5 Signaling in VSMC

**Figure 5:**
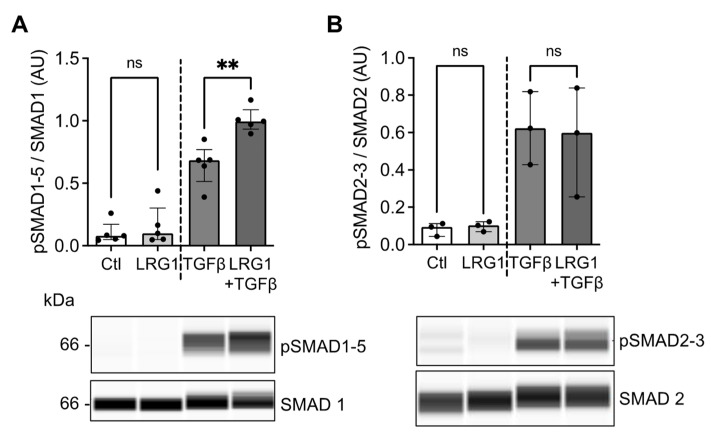
Effect of LRG1 on TGFβ-induced SMAD signaling. (**A**,**B**) Quantification and representative capillary Western blots of pSMAD1-5/SMAD1 and (**B**) pSMAD2-3/SMAD2 in MOVAS treated with 20 μg/mL LRG1 with or without 10 ng/mL TGFβ for 1 h. *n* = 3–5 independent experiments/group; ns: non significative; ** *p* < 0.01 via Mann–Whitney U test. Data are presented as median with lower and upper quartiles.

It has been shown that LRG1 modulates TGFβ signaling [20,21,24]. Furthermore, TGFβ signaling has been extensively described as being involved in the process of vascular calcification [25,26]. We, therefore, investigated whether LRG1 could modulate TGFβ signaling pathways in VSMC. In the absence of TGFβ, treatment with LRG1 did not trigger the phosphorylation of the SMAD1/5 and SMAD2/3 proteins in MOVAS (Figure 5A,B). However, when MOVAS were treated with LRG1 in the presence of TGFβ, we observed a significant increase in the phosphorylation of SMAD1/5 compared to treatment with TGFβ alone (Figure 5A). On the other hand, TGFβ-induced phosphorylation of SMAD2/3 was not modulated via LRG1 (Figure 5B). These results suggest that LRG1 can potentiate TGFβ-induced SMAD1/5 signaling in VSMC. 

## 3. Discussion

It is known that plaque complications greatly affect plaque stability [3]. While the causal role of calcification in plaque rupture remains under evaluation, it has been established that calcification serves as a predictive marker of arterial stiffness [11,27], which is associated with the development of cardiovascular complications [12,28]. Understanding the molecular mechanisms involved in vascular calcification is therefore crucial in identifying and proposing novel therapeutic strategies. The current study establishes a possible novel function for the glycoprotein LRG1 in the pathogenesis of plaque complications and arterial calcification.

LRG1 is a glycoprotein that has been identified in plasma [29], and, as such, circulating LRG1 has been extensively described as upregulated in numerous pathological conditions, including cardiovascular disease [19,22,23,30]. Under physiological conditions, LRG1 appears to be primarily synthetized by hepatocytes [31] and neutrophils [32]. In addition, its expression appears to be regulated under pathological conditions. LRG1 is upregulated in endothelial cells and various tissue microenvironments, including epithelial cells, myeloid cells, and fibroblasts during inflammatory conditions [33], indicating its potential involvement in inflammatory diseases. The various pathological roles of LRG1 have resulted in the development of new therapeutic approaches that target LRG1 inhibition. Currently, the possibility of an LRG1 blockade is under evaluation in humans for its anti-angiogenic effects [34,35]. However, LRG1 expression in atherosclerosis remains unexplored. 

Our findings reveal the accumulation of LRG1 in the neointima of complicated atherosclerotic plaques in both human and murine contexts. LRG1 accumulation was identified preferentially around calcified lesions, which correspond to the necrotic core area that is rich with calcium crystals. To our knowledge, we are the first to report the accumulation of LRG1 in the neointima. Since different studies reported the secretion of LRG1 under inflammatory stimuli, we investigated a possible local expression of this glycoprotein in vascular cells under stimulation with inflammatory mediators. We demonstrated that endothelial cells, but not VSMC, can produce LRG1 under cytokine stimulation. This suggests that endothelial cells may be responsible for LRG1 secretion into the subendothelial space. Another possible explanation is that under high blood pressure, LRG1 could accumulate in the arterial wall due to convection forces associated with the increased permeability of blood microvessels and endothelium [36]. However, these hypotheses do not provide an explanation for the specific accumulation of LRG1 in the necrotic core area. An additional possibility is that LRG1 originates from vesicles derived from inflammatory cells present in the necrotic core [37] since LRG1 has been described to be produced by macrophages and neutrophils [32,37,38].

The specific localization of LRG1 within calcified regions of atherosclerotic plaques led us to investigate the potential causal link between LRG1 and arterial calcification. Notably, our in vitro findings demonstrate the capacity of LRG1 to induce the trans-differentiation of VSMC into osteoblast-like cells. Although extensive research has been conducted regarding the role of LRG1 on angiogenesis, investigations into its other potential functions remain limited. A few studies have suggested the role of LRG1 in fibrosis in various organs, such as the skin, lungs, and kidneys [24,39,40]. These effects are largely attributed to the modulation of TGFβ signaling via the binding of LRG1 to endoglin. While endoglin is predominantly expressed in endothelial cells, its expression has also been demonstrated in VSMC [41,42]. However, the impact of endoglin expression on VSMC function varies depending on the developmental or pathological context. Tian et al. [41] demonstrated that endoglin expression in VSMC facilitates their recruitment via endothelial cells by promoting VSMC migration and spreading on endothelial cells. In contrast, Ma et al. [42] revealed that endoglin is overexpressed in VSMC after arterial injury and is crucial for the TGFβ-induced inhibition of VSMC migration. Our findings present the first evidence that LRG1 potentiates VSMC calcification and increases osteoblastic trans-differentiation. Further experiments need to be performed to better characterize the involvement of endoglin in these processes.

The Involvement of SMAD signaling pathways via TGF family receptors in smooth muscle cell calcification has been extensively studied in the past [43,44]. While SMAD1/5/8 is classically activated via BMP2 in VSMC and leads to vascular calcification [44,45,46], SMAD2/3 has been directly linked to TGFβ activation and promotes smooth muscle cell differentiation [47,48]. Interestingly, we showed that LRG1 enhanced the activation of the TGFβ-induced SMAD1/5 signaling pathway in VSMC without affecting the SMAD2/3 axis. In line with our findings, it has also been demonstrated that LRG1 acts as a modulator of TGFβ signaling pathways in endothelial cells by promoting the SMAD1/5 pathway via the recruitment of endoglin and resulting in cell proliferation [20,21]. Our results indicate that LRG1 can activate common signaling pathways in endothelial and VSMC, each leading to distinct functions. The use of SMAD inhibitors could be used in further studies to establish that this pathway is indeed responsible for mediating the effect of LRG1 in VSMC trans-differentiation and calcification. 

Previous work reported that LRG1 levels were associated with disease progression. For example, in mice and humans presenting diabetic kidney disease, LRG1 expression is increased in glomerular endothelial cells [20,49,50], contributing to microvascular instability. According to these data, *Lrg1* deletion protected mice from streptozotocin-induced diabetic kidney disease by decreasing angiogenesis, reducing podocyte foot process effacement, and improving kidney function. In line with our observations, this effect was due to a redirection of TGFβ signaling towards a SMAD1/5 signaling pathway [20]. While, in most cases, high levels of LRG1 have been associated with disease progression, its contribution to cardiovascular disease remains unclear, with both protective and pathological properties being reported. In a murine model of myocardial infarction, LRG1 levels were increased in the early phases post-infarct and decreased in the late fibroproliferative phases [51]. The deletion of *Lrg1* in this model leads to impaired perfusion, increased matrix deposition, and impaired cardiac function [51]. It is, in fact, possible that LRG1 is initially involved in a healing process, leading to increased vascularization and cardiomyocyte survival via the activation of autophagy, but that this deregulated process may subsequently lead to cardiomyocyte death and increased fibrosis [52,53,54]. So, while the possibility of using LRG1 as a biomarker in cardiovascular pathologies seems clear, its causal role in these pathologies remains to be elucidated, particularly regarding the complications of atherosclerosis. The use of LRG1^−/−^ mice injected with gain-of-function mutant PCSK9 adeno-associated virus vector capable of leading to complicated atherosclerotic plaques presenting calcifications [55] could be used in further studies to validate this hypothesis.

Our findings offer novel perspectives on the function of LRG1 in the vascular wall and present new opportunities for employing innovative LRG1-targeting therapies to decelerate the calcification process, which continues to pose a significant challenge to individuals with diabetes and chronic renal failure.

## 4. Materials and Methods

### 4.1. Mouse Model

Ten-week-old male and female ApoE−/− mice on a C57BL/6J background were fed normal chow (CD) or Western diet (WD, 42% from fat, TD88137, ENVIGO, Gannat, France) and water ad libitum throughout this study. Animal experiments were performed in compliance with the guidelines on animal experimentation and with a French Ministry of Agriculture and Food license (2020121714324566). The mice were sacrificed at 37 weeks old after 27 weeks of diet. After PBS flushing, the entire aorta was isolated and fixed in 4% PFA before en face aorta analysis. Aortic roots were embedded in OCT and frozen at −80 °C.

### 4.2. Histological Analysis and Immunohistochemical Staining

Ten μm serial sections were prepared throughout 1 mm of aortic sinus region starting from the appearance of the first valve. The cryosections were dehydrated at room temperature for 10 min before being fixed either in 10% formaldehyde or 4% paraformaldehyde. For the Oil Red O staining (01391-500 mL, Sigma, Saint Quentin, France), the cryosections were washed with 30% isopropanol solution before being stained in 0.1% staining solution and counterstained with hematoxylin. For the Alizarin staining, the cryosections were stained in 2% Alizarin solution pH 4.2 (A5533, Sigma, Saint Quentin, France). For LRG1 immunostaining, cryosections were saturated with 1% BSA solution before being incubated for 1 h with the anti-LRG1 antibody solution (diluted 100 e, PA5-96832, Fisher Scientific, Illkirch, France). Sections were then incubated with secondary antibody solution before being detected with DAKO K3468 and counterstained with hematoxylin. The human artery paraffin sections were donated by Anne Nègre-Salvayre (provided by the Cardiovascular Surgery Department, CHU Toulouse, France, Pr. Y. Glock between 2005 and 2008) [56] and stained with an anti-LRG1 antibody solution (diluted 100 e, PA5-23266, Illkirch, France). 

### 4.3. Cell Culture

MOVAS (CRL-2797™, ATCC, Molsheim, France) were cultivated from passages 3 to 6 in Dulbecco′s Modified Eagle′s Medium (DMEM) with 4.5 g/L glucose (D0822, Sigma, Saint Quentin, France) supplemented with 10% FBS (A31608-01, GIBCO, Fisher Scientific, Illkirch, France), 1% penicillin/streptomycin (P0781, Sigma, Saint Quentin, France), and 0.4 mg/mL G418 (G8168, Sigma, Saint Quentin, France) at 37 °C with 5% CO2. Primary human aortic smooth muscle cells isolated from the ascending aorta or the aortic arch (hASMCs) (CC-2571, Lonza, Basel, Switzerland) were maintained at 37 °C in a humidified atmosphere with 5% of CO2in SMGM TM-BulletKit (CC-3182, Lonza, Basel, Switzerland) until confluence, as previously described [57]. To induce cell calcification, the cells were treated for 6 days with 2.6 mM phosphate (Na2HPO4 71629, Fisher Scientific, Illkirch, France and NaH2PO4 33662, Fisher Scientific, Illkirch) with/without 20 μg/mL of LRG1 (7890-LR-MTO, Bio-techne, Rennes, France). Fresh media with agents was added every 2–3 days. The cells were treated with 10 ng/mL of TNFα or 4 ng/mL IL1β for 24 h or 48 h for measurement of LRG1 expression, with 10 ng/mL TGFβ for 1 h for signaling, or with calcification medium for 72 h for expression of osteochondrogenic markers and 6 days for Alizarin staining.

### 4.4. Quantification of Calcium Deposition

Cells were decalcified with 0.1 M of HCl, and the calcium content was measured with QuantiChrom Calcium Assay Kit (BioAssay Systems, Gentaur, Paris, France) according to the manufacturer’s directions. Calcium content was normalized to total protein concentration measured using Bradford assay.

### 4.5. Real-Time Quantitative PCR

Total RNA was extracted from cell lysates with Trizol reagent (79306, QIAzol^®^, Qiagen, Marseille, France) according to the manufacturer’s directions. Reverse transcription of 500 ng RNA was performed using Revertaid H minus (EP0451, Fisher Scientific, Illkirch, France). The cDNA was amplified via RT-qPCR using SsoFast™EvaGreen^®^ Supermix (#172-65204, Bio-rad, Marne, France) with the following primer sequences: Spp1 m (F) 5′-CCCGGTGAAAGTGACTGATT-3′ and (R) 5′-TTCTTC AGAGGACACAGCATTC-3′, Runx2 m (F) 5′-CGTGTCAGCAAAGCTTCTTTT-3′ and (R) 5′-GGCTCACGTCGC TCATCT-3′, Ankh m (F) 5′-CCATTGTCAACCTCTTCGTG-3′ and (R) 5′-GGGTAGGTGG CTGTCAGAAT-3′, LRG1 m (F) 5′-CCATGTCAGTGTGCAGATTC-3′ and (R) 5′-AAGAGTGAGAGGTGGAAGAG-3′, LRG1 h (F) 5′-GTTGGAGACCTTGCCACCT-3′ and (R) 5′-GCTTGTTGCCGTTCAGGA-3′, Rpl13a (F) 5′-CAT GAGGTCGGGTGGAAGTA-3′ and (R) 5′-GCCTGTTTCCGTAACCTCAA-3′. All RT-qPCR were performed on a ViiA™ 7 Real-Time PCR System (Applied Biosystems, Fisher Scientific, Illkirch, France), in duplicate, and were normalized with relative mRNA expression of Rpl13a and the control group (2^−ΔΔCt^).

### 4.6. Capillary Western Blot Analysis

Capillary Western blot analyses were performed using the ProteinSimple Wes^®^ System (Bio-techne, Rennes, France). Proteins were loaded into Wes capillaries, and columns were probed with anti-mouse antibodies for pSMAD2 (diluted 20 e with samples diluted at ¼, 3108 Cell signaling), SMAD2/3 (diluted 100 e with samples undiluted, 610,842 BD Science), pSMAD1/5 (diluted 50 e with samples undiluted, 9516 Cell signaling), SMAD1 (diluted 100 e with samples diluted at ½, 9743 Cell signaling). The levels of specific proteins were determined using the Wes capillary protein detection system (ProteinSimple, Bio-techne, Rennes, France) according to the manufacturer’s directions.

### 4.7. Statistical Analysis

All data were presented as mean ± SEM. All statistical analyses were performed with GraphPad Prism 8. Data normality was analyzed using the Shapiro–Wilk normality test. For normally distributed data, statistical analysis was performed with two-sided Student’s t-test for comparison between two groups, a One-way ANOVA test followed by Tukey’s multiple comparisons for comparisons between three groups or more. The Mann–Whitney U test or Kruskal–Wallis’s test with Dunn’s multiple comparisons was used for non-normally distributed data. A *p* < 0.05 was considered as significant.

## Data Availability

Data will be made available upon reasonable request.
